# A High-Repeatability Three-Dimensional Force Tactile Sensing System for Robotic Dexterous Grasping and Object Recognition

**DOI:** 10.3390/mi15121513

**Published:** 2024-12-20

**Authors:** Yaoguang Shi, Xiaozhou Lü, Wenran Wang, Xiaohui Zhou, Wensong Zhu

**Affiliations:** School of Aerospace Science and Technology, Xidian University, Xi’an 710071, China; shiyg@xidian.edu.cn (Y.S.); 15029675366@163.com (W.W.); 22131214128@stu.xidian.edu.cn (X.Z.); zhuwensong73@163.com (W.Z.)

**Keywords:** three-dimensional force tactile sensing, high-repeatability feature, robotic dexterous fingertip, object recognition

## Abstract

Robotic devices with integrated tactile sensors can accurately perceive the contact force, pressure, sliding, and other tactile information, and they have been widely used in various fields, including human–robot interaction, dexterous manipulation, and object recognition. To address the challenges associated with the initial value drift, and to improve the durability and accuracy of the tactile detection for a robotic dexterous hand, in this study, a flexible tactile sensor is designed with high repeatability by introducing a supporting layer for pre-separation. The proposed tactile sensor has a detection range of 0–5 N with a resolution of 0.2 N, and the repeatability error is as relatively small as 1.5%. In addition, the response time of the proposed tactile sensor under loading and unloading conditions are 80 ms and 160 ms, respectively. Moreover, a three-dimensional force decoupling detection method is developed by distributing tactile sensor units on a non-coplanar robotic fingertip. Finally, using a backpropagation neural network, the classification and recognition processes of nine types of objects with different shapes and categories are realized, achieving an accuracy higher than 95%. The results show that the proposed three-dimensional force tactile sensing system could be beneficial for the delicate manipulation and recognition for robotic dexterous hands.

## 1. Introduction

The state-of-the-art robotic sensing methods using vision [1] or audio [2] devices can detect environmental information, but it is challenging to extract detailed features of the target objects through contactless measurement. Therefore, to explore unknown environments effectively and realize more delicate manipulation, the current research employs robotic dexterous hands with perception functions, which play an increasingly important role in the robotic field [3,4,5,6]. Tactile sensors can provide certain environmental information, such as the contact state, position, and force intensity during the object manipulation process, which is of great significance for realizing the dexterous manipulation of a robot’s dexterous hands [7,8,9,10]. Recent studies have proposed various tactile sensors based on the sensing mechanisms using the piezoresistive [11,12,13], capacitive [14,15], triboelectric [16,17,18], electret [19], and magnetic [20] properties. Among them, the piezoresistive mechanism is one of the most used due to its simple configuration and measurement circuit, which makes it a promising tactile detection methods.

The existing flexible piezoresistive tactile sensors can be roughly divided into the following two types based on their sensing mechanisms: the bulk resistance sensing type and the contact resistance sensing type. In the piezoresistive tactile sensors based on bulk resistance sensing, the solid or porous piezoresistive material is fixed to the electrodes directly by soldering, curing, or pasting. Therefore, when an external force is applied to this type of sensor, the bulk resistance of the piezoresistive material varies accordingly. For instance, Cai et al. [21] fabricated a piezoresistive elastomer foam based on the polydimethylsiloxane/multi-walled carbon nanotube (PDMS/CNT) by directly introducing thermo-expandable microspheres (EMs). The piezoresistive tactile sensor has a detection range of 15 kPa–280 kPa, but a bulk resistance sensing type tactile sensor with a solid material usually has relatively low sensitivity due to the limited elasticity, and the porous material suffers from the relatively thicker volume. In contrast, in a piezoresistive tactile sensor of the contact resistance sensing type, there are variations in the contact resistance between the multi-layer piezoresistive materials or between the piezoresistive material and the electrodes due to the coordinated pressure change. Yue et al. [22] developed a piezoresistive sensor with the MXene-coated sponge material, which could detect pressure in a range of 0~18.56 kPa, with a maximum sensitivity of 442 kPa^−1^. Shi et al. [23] designed a flexible piezoresistive pressure sensor using a multi-layer microstructured composite material, whose pressure detection range and sensitivity were 0–583 kPa and 7.66 kPa^−1^, respectively. However, the piezoresistive tactile sensors based on the contact resistance usually face the problem of the initial resistance drift [24] caused by the complex surface microstructures and uneven dispersion of the nanoparticles in the composite materials. The limited repeatability of piezoresistive tactile sensors restricts their application in robotic dexterous hand grasping and recognition tasks.

Moreover, there has been an urgent demand for developing a three-dimensional (3D) force perception for a robotic dexterous hand. The 3D force decoupling of flexible tactile sensors can be realized using a decoupled structural design or material characteristics [25,26,27,28] and by employing deep learning [29,30,31]. Among these methods, the decoupled structural design is easy to realize for a tactile sensor, but this design is difficult to miniaturize and integrate into a robotic dexterous hand fingertip due to the relatively complex encapsulation and electrodes. However, using deep learning-based methods, such as neural networks, the training of a flexible tactile sensor array with a more integrated structural design can be achieved. Still, this process requires a large amount of training data, and the implementation procedure is relatively complex.

Aiming to solve the problem of initial resistance drift of piezoresistive tactile sensors, in this study, a flexible tactile sensor is proposed with high repeatability by introducing the supporting layer for pre-separation. The experimental characterization and the comparison are conducted. In addition, a non-planar tactile sensing system for three-dimensional force decoupling is developed for robot dexterous fingertips. Finally, by using a neural network for the training, the grasped objects with different shapes and material characteristics are identified and classified by the proposed 3D force sensing system.

## 2. Materials and Methods

### 2.1. Proposed Sensor Design and Operational Principle

**Figure 1:**
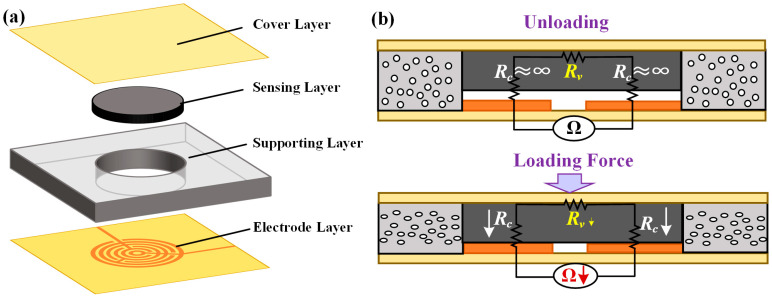
The proposed flexible tactile sensor: (**a**) the schematic diagram; (**b**) the sensing principle.

The structural design of the proposed flexible tactile sensor unit is shown in Figure 1a. The sensor unit consists of the following four main components: a cover layer, a supporting layer, a sensing layer, and a bottom electrode layer. In the bottom electrode layer, the copper interdigital electrodes are made on the polyimide (PI) film substrate. The supporting layer contains an electronic crosslinked polyethylene (IXPE) layer with porous microstructures. In the middle of the supporting layer, there is a cylindrical hollow structure set, and the piezoresistive sensing layer made of conductive carbon black in rubber is placed right through the hole and above the copper interdigital electrodes. The cover layer fixes the supporting layer and the upper surface of the sensing layer.

The functions of the supporting layer lie in pre-separation and the assisted recovery of the sensing layer. For pre-separation, the supporting layer is designed to be a little thicker than the sensing layer, ensuring that there is no contact between the sensing layer and the interdigital electrodes during unloading, which can lead to an approximately open circuit, with an extremely large initial resistance for a tactile sensor; this can further avoid the inconsistent initial resistance values of different units in the sensing arrays. For assisted recovery, the elastic modulus of the IXPE supporting layer is set to be smaller than that of the rubber-like sensing layer. Namely, when the sensor is under loading conditions, the supporting layer can be compressed along with the sensing layer, making contact with the electrodes. Therefore, after removing the loading force, the thicker supporting layer can help the sensing layer recover to its initial state, which can further improve the repeatability of the sensor.

The operational principle of the proposed flexible tactile sensor is based on the change in the contact resistance between the sensing layer and the interdigital electrodes. As shown in Figure 1b, the total resistance of the sensor (*R*) consists of the bulk resistance (*R_V_*) of the sensing layer and the contact resistance (*R_c_*). During the unloading state, the initial value of *R_c_* is extremely large. However, when the force is applied to the tactile sensor, the change in the *R_c_* value will be much larger than *R_V_*, and the total resistance of the sensor will decrease.

### 2.2. Finite Element Modeling

**Figure 2:**
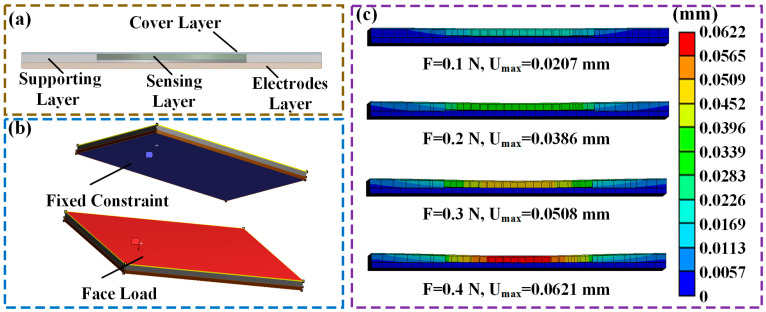
(**a**) The cross-sectional view of the proposed flexible tactile sensor unit’s 3D model; (**b**) the boundary conditions in the modeling process; (**c**) the finite element analysis results obtained under the force of 0.1 N–0.4 N.

To investigate the effect of the supporting layer on the pre-separation performance, in this study, the corresponding flexible tactile sensor is modeled and analyzed using the finite element modeling method through COMSOL Multiphysics. The cross-section view of the 3D-modeled tactile sensor is illustrated in Figure 2a. Aiming to fix the sensing layer, in this study, a cylindrical hollow structure is used with a radius of 2.5 mm and is set in the middle of the supporting layer. The material parameters of the sensing layer and IXPE-made supporting layer are determined by an experiment analysis. Due to the small pressure range, the components of the sensing and supporting layers are simplified to linear elastic materials. Further, as the electrode layer is mounted to the fingertips in application, the electrode layer is set as a rigid metal (steel). The material parameters used in the finite element modeling are listed in Table 1. The boundary conditions of the modeling process are shown in Figure 2b.

In the finite element analysis, the bottom surface of the electrode layer was set as a fixed constraint, and a force of 0.1 N–0.4 N was loaded on the top surface of the cover layer. The surface between the sensing and supporting layers was set as a friction contact with a friction coefficient of 0.2. The contact types of other surfaces were set as binding contact. Finally, the 3D model of the flexible tactile sensing was meshed into 2726 grids.

The results of the finite element analysis are illustrated in Figure 2c. In the initial phase, due to the pre-separation effect of the supporting layer, the distance between the sensing layer and the electrode layer was 0.05 mm. In addition, it was observed that when the normal force increased from 0.1 N to 0.2 N, the maximum displacement of the sensing layer was always smaller than 0.05 mm, making the output resistance of the sensor extremely large. When the pressure increased above 0.3 N, the maximum deformation displacement of the sensing layer was 0.062 mm. Accordingly, the sensitive layer was in contact with the electrode layer, and the contact resistance decreased sharply. Based on the simulation results, the supporting layer could provide pre-separation for the sensor, and thus avoiding the sensor’s initial resistance drift.

## 3. Experimental Parameters

### 3.1. Fabrication Process

The tactile sensor unit was fabricated using flexible materials. The cover and supporting layers were made of 20 µm-thick PI tape (3J Tape Co., Ltd., Dongguan, China) and 150 µm-thick IXPE foam (Shenzhen Yizhong Technology Co., Ltd., Shenzhen, China), respectively. The cylinder piezoresistive sensing layer had a diameter of 2.5 mm and a thickness of 0.1 mm. The sensing layer in the developed tactile sensor was made of PE particles added with conductive carbon black and metallocene blown molding. The electrode layer was fabricated using the traditional flexible printed circuit board (FPCB) process, and the circular interdigital sensor electrodes with a diameter of 2.5 mm were fabricated on the PI substrate (Shanghai Youren Electronic Technology Co., Ltd., Shanghai, China). The PI tape was cut and fixed to the IXPE foam with hollows and the piezoresistive sensing arrays by sticking the top surface of them. The edge of FPCB electrodes was fixed to the PI tape by connecting the remaining part of the PI tape. As the diameter of the sensing layer is limited, the tactile sensor could be properly fixed. Thus, the piezoresistive sensing layer was kept separating to the electrodes after fabrication. The interdigital electrode with 60% occupied area was selected by experimental test according to the resistance variation range of the sensor. The fabricated tactile sensor features an overall thickness of about 0.4 mm.

### 3.2. Experimental Set-Up

**Figure 3:**
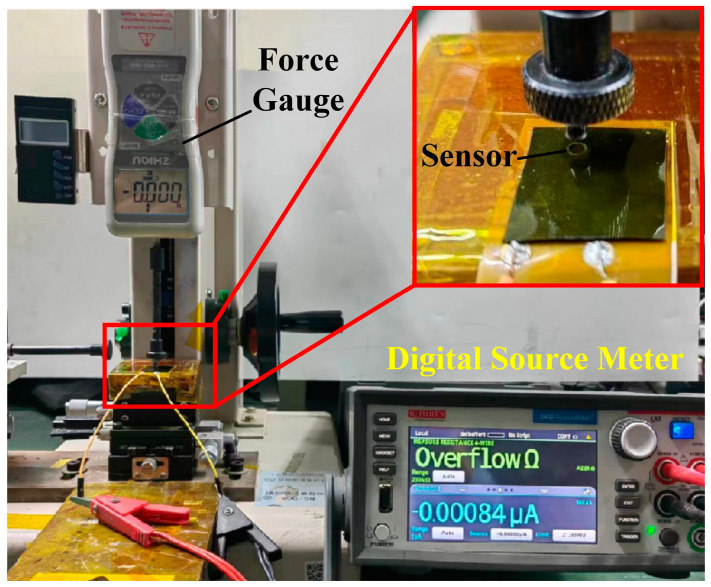
Photograph of the property-characterization experimental setup.

To characterize the properties of the fabricated tactile sensor unit, in this study, the experimental setup was constructed in a laboratory, as shown in Figure 3. A customized loading head was installed in the push–pull force gauge (DS2-50N, Dongguan Zhiqu Precision Instrument Co., Ltd., Dongguan, China), which was consistent with the size of the tactile sensor unit. The digital source meter (2450 SMU, Tektronix Inc., Beaverton, OR, USA) was used to record changes in the output resistance of the fabricated flexible tactile sensor unit under different normal loading forces with the sampling frequency is 50 Hz.

## 4. Results and Discussion

**Figure 4:**
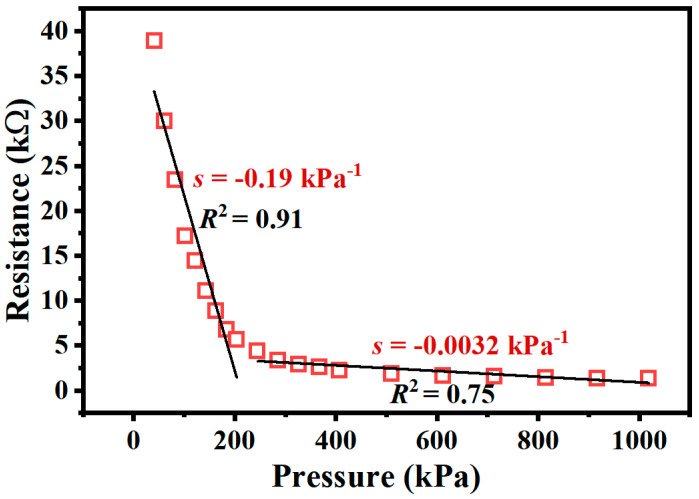
The relationship between the force intensity and the resistance of the flexible tactile sensor unit.

The relationship between the force and the resistance of the fabricated flexible tactile sensor unit is shown in Figure 4, where it can be observed that the output resistance of the sensor unit was very large and close to that of an open circuit under the initial unloading condition. The output resistance rapidly decreased after the force was applied. According to the FEM results, the LOD of the tactile sensor is about 0.2 N. As the normal force increased from 0.2 N to 5 N, the output resistance of the tactile sensor unit decreased from 38.5 kΩ to 1.2 kΩ. Considering the area of the piezoresistive sensing layer, the pressure detection range of the proposed flexible tactile sensor unit was from 40.744 kPa to 1.019 MPa.

The sensitivity (*s*) of the proposed piezoresistive tactile sensor unit represented the ratio of the output resistance variation to the applied pressure increment, and it was calculated by the following:

(1) $$s=\frac{\delta (∆R/R)}{\delta P}$$

where ∆*R* represents the change in output resistance in the full pressure detection range; *R* is the resistance of the tactile sensor at the maximum normal pressure; and *δP* indicates the normal stress variation. As shown in Figure 4 it can be observed that the relationship between the pressure and the resistance is nonlinear. The tactile sensor featured a better linearity in the range of 0–1 N with a sensitivity of −0.19 kPa^−1^ and a coefficient of determination (*R*^2^) of 0.91. However, in the detection range of 1–5 N, the sensitivity and *R*^2^ reduce to −0.0032 kPa^−1^ and 0.75, respectively.

**Figure 5:**
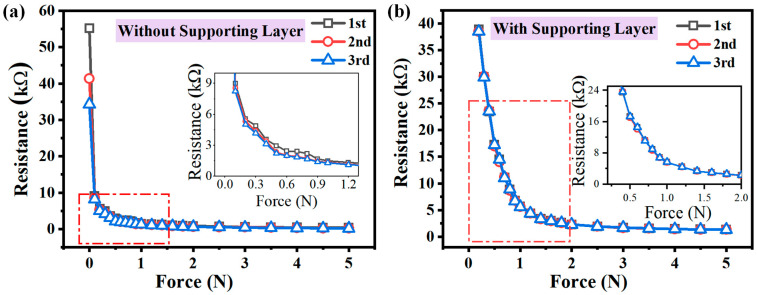
The relationship between the force and the proposed flexible tactile sensor unit’s resistance: (**a**) without the supporting layer; (**b**) with the supporting layer.

For the experimental investigation of the supporting layer’s effect on the proposed sensor unit’s performance, different piezoresistive sensor units with and without the supporting layer were fabricated and characterized. The feature comparison of the two types of sensor units is shown in Figure 5, where it can be observed that the initial resistance varied in three times under loading for the sensor unit without the supporting layer, as shown in Figure 5a. On the contrary, the initial resistance maintained stability for the sensor unit with the supporting layer, as illustrated in Figure 5b. Moreover, the output resistance value of the sensor unit without the supporting layer showed inconsistency for different loading procedures, which was because the piezoresistive sensing layer was directly attached to the interdigital electrodes by the preload force of the cover layer. However, after introducing the supporting layer, the sensor’s repeated test results showed better consistency. The repeatability error could be calculated by the following:

(2) $$\delta_{R}=\frac{\left|Z\delta_{max}\right|}{Y_{FS}}\times 100\%$$

where $\delta_{R}$ is the repeatability error; *Z* is the fiducial probability, which is set as 99.73%; *Y_FS_* is the full detection range of the tactile sensor unit; and $\delta_{max}$ is the standard deviation, which could be calculated by the following:

(3) $$\delta_{max}=\sqrt{\frac{1}{N-1}\sum_{i=1}^{N}(F_{i}-\bar{F}{)}^{2}}$$

where *N* is the number of the experimental test and *F_i_* is the test result in the ith time and average test data, respectively. Thus, the repeatability error of the proposed tactile sensor unit is 1.5%. Comparing to other common taxel-based fingertip setups, the repeatability error is relatively low for the robotic tactile perception. For instance, Choi et al. [32] developed a soft three-axis force sensor based on radially symmetric pneumatic chambers. The repeatability error was ±2.26%, ±4.76%, and ±2.40% for the *x*-, *y*-, and *z*-axes, respectively. Kaidarova et al. [33] developed a flexible Hall sensor made of laser-scribed graphene with a standard deviation of σ ± 0.002 N. The results showed that the proposed tactile sensor could be utilized for repeated grab recognition.

**Figure 6:**
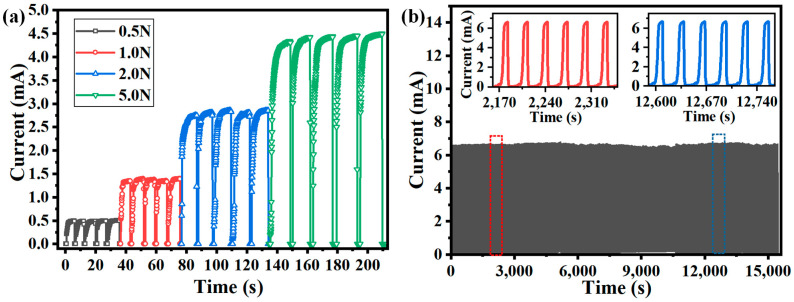
(**a**) The response of the sensor unit subjected to the repeated force of 0.5 N, 1 N, 2 N, and 5 N; (**b**) the cyclic response of the tactile sensor unit under loading for 500 cycles.

**Figure 7:**
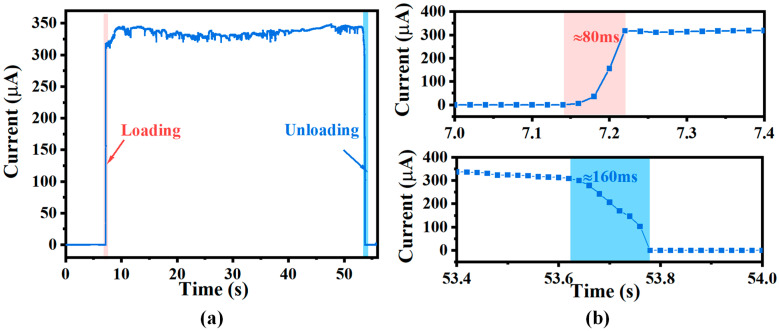
(**a**) The dynamic response of the sensor under the applied normal force of 0.5 N; (**b**) the response time under loading and unloading conditions.

Further, the tactile sensor unit was repeatedly loaded five times using the different forces of 0.5 N, 1 N, 2 N, and 5 N to investigate the repeatability of the tactile sensor. The digital source meter (2450 SMU) was used to measure the current change under a constant DC voltage of 10 V with a sampling frequency of 50 Hz. As shown in Figure 6a, the tactile sensor unit showed good repeatability in detecting different normal forces. Moreover, the cyclic characteristics of the tactile sensor unit were investigated using various normal forces by a material testing machine (TY8000, Jiangsu Tian Yuan Test Instrument, Yangzhou, China). As shown in Figure 6b, after 500 cycles of loading, the tactile sensor unit still had good cycle characteristics, indicating that the flexible tactile sensor with the supporting layer could promote high reliability and durability in the robotic prosthetic hand tactile sensing applications. Furthermore, the dynamic response characteristics of the output resistance of the flexible tactile sensor unit were investigated by applying a normal pressure of 0.5 N, as shown in Figure 7. The results demonstrated that the instantaneous loading and unloading times were 80 ms and 160 ms, respectively. Finally, the developed tactile sensor unit had a relatively fast response time and could be suitable for dexterous hand-grasping applications.

## 5. Application Prospects

### 5.1. Robotic Dexterous Hand Integration

**Figure 8:**
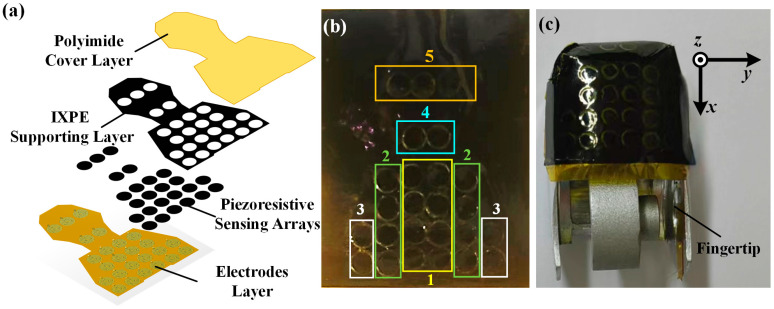
(**a**) The schematic diagram of the proposed tactile sensor array; (**b**) the photograph of the sensor array prototype; (**c**) the fingertip with the non-coplanar sensor array mounted.

Due to the small total thickness of the proposed flexible tactile sensor unit of less than 1 mm, the piezoresistive sensing layer could hardly realize the shear force detection. Therefore, it is necessary to develop a method that can meet the requirements for 3D force detection of a robotic dexterous hand. To this end, in this study, the dexterous fingertips were designed as multiple planes at an angle to each other, and the tactile sensor array could distribute on these non-coplanar planes. The force detection range and resolution of each plane were related to the number of distributed tactile sensor units. The structural design of the tactile sensor array is shown in Figure 8a, where it consisted of a cover layer, a supporting layer, piezoresistive sensing arrays, and an electrode layer. In the supporting layer, there were cylindrical hollow structures, which corresponded to the piezoresistive sensing array and the interdigital electrodes. As shown in Figure 8b, there were 25 circular tactile sensor units with a diameter of 2.5 mm distributed on seven regions, which corresponded to the non-coplanar planes of the robotic dexterous hand fingertip. In particular, the non-coplanar planes were classified into one normal force detection plane and six shear force detection planes, and the shear force detection planes were further divided into four types. According to the structural design of the robot’s dexterous fingertip, the angles between each section were calculated. As shown in Figure 8c, Plane 1 was perpendicular to the fingertip, and it was set as the *z*-axis direction; Plane 5 was perpendicular to the top of the fingertip, and it was set as the *x*-axis direction; Planes 2–4 were perpendicular to the fingertip side and set as the *y*-axis direction. Thus, the force detection capability of each plane was obtained by the following:

(4) $$\left\{\begin{array}{c} F_{1}=8F_{s}\\ F_{2}=4F_{s}\cos\left(77.25{}^{\circ}\right)\\ F_{3}=2F_{s}\cos\left(62.09{}^{\circ}\right)\\ F_{4}=2F_{s}\cos\left(46.47{}^{\circ}\right)\\ F_{5}=3F_{s}\cos\left(9.03{}^{\circ}\right) \end{array}\right.$$

where *F*_1_, *F*_2_, *F*_3_, *F*_4_, and *F*_5_ are the resultant forces for different non-coplanar planes, and *Fs* is the detected force value of each tactile sensor unit.

As mentioned above, the proposed tactile sensor unit had a force detection range of 0.2 N–5 N. According to Equation (3), *F*_1_ features a normal force detection range of 1.6~40 N, *F*_2_ features a shear force detection range of 0.17~4.41 N on the *y*-axis, *F*_3_ features a shear force detection range of 0.18~4.68 N on *y*-axis, *F*_4_ features a shear force detection range of 0.27~6.89 N on *x*-axis, and *F*_5_ features a shear force detection range of 0.59~14.81 N on *x*-axis. Thus, the detection ranges of the normal and shear forces were about 0–20 N and 0–10 N, respectively.

### 5.2. Object Hardness Recognition

**Figure 9:**
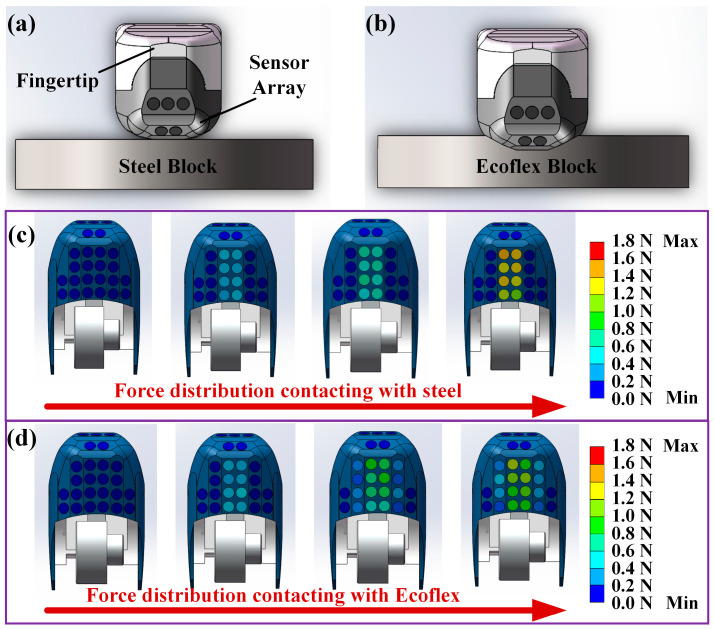
The schematic diagram of the robotic dexterous hand fingertip with the sensor array contacting: (**a**) the rigid steel block; (**b**) the flexible Ecoflex block. The heat map of the force detection of the fingertip with the sensor array contacting: (**c**) the rigid steel block; (**d**) the flexible Ecoflex block.

To simulate the process of rigid object grasping for robotic dexterous fingertips, in this study, the force information was obtained experimentally and recorded on each tactile sensor unit in the following four states: (1) before grasping (the dexterous hand fingertips were not in contact with the grasped object); (2) the initial grasping stage (there was slippage between the dexterous fingertips and the grasped object); (3) the grasping stable stage (the dexterous finger grasped the object stably, and there was no slippage); (4) the gripping stage (the dexterous finger grasped the object stably and then increased the grasping force). When keeping the grasping posture, the detected force was recorded for 5 s after the object state became stable. The schematic diagram of the posture of the fingertips grasping the rigid object is displayed in Figure 9a. The grasped rigid object was a cylinder made of steel with a radius of 3 cm and a thickness of 1.5 cm. Based on the changes in the normal force value of each tactile sensor unit, the force distribution heat map of the fingertip was determined, as shown in Figure 9c. During the process of grasping the rigid steel block, only the eight tactile sensor units on the normal force detection plane contacted the object, while the pressure values of the remaining units were always zeros.

The schematic diagram of the process posture when the fingertip grasped an elastic object is shown in Figure 9b. The elastic block made of Ecoflex (Smooth-On, Inc., Macungie, PA, USA) had an elastic modulus of approximately 125 kPa, which could deform during the fingertip contact process. According to the changes in the force values of each tactile sensor unit, the force distribution in the four states was determined, as illustrated in Figure 9d. When the dexterous fingertip contacted the Ecoflex block at the beginning of the grasping process, only the eight tactile units on Plane 1 responded to the applied force. As the force applied increased, the Ecoflex block deformed further, and the tactile units on the other shear force detection planes of the fingertip could detect the force. Therefore, when the dexterous hand grasped the elastic and deformable objects, the number and distribution of tactile sensor units in contact changed with the grasping force intensity. Thus, the object hardness recognition could be realized using the proposed tactile sensing array mounted on the fingertip for 3D force detection.

### 5.3. Grasping Object Recognition

**Figure 10:**
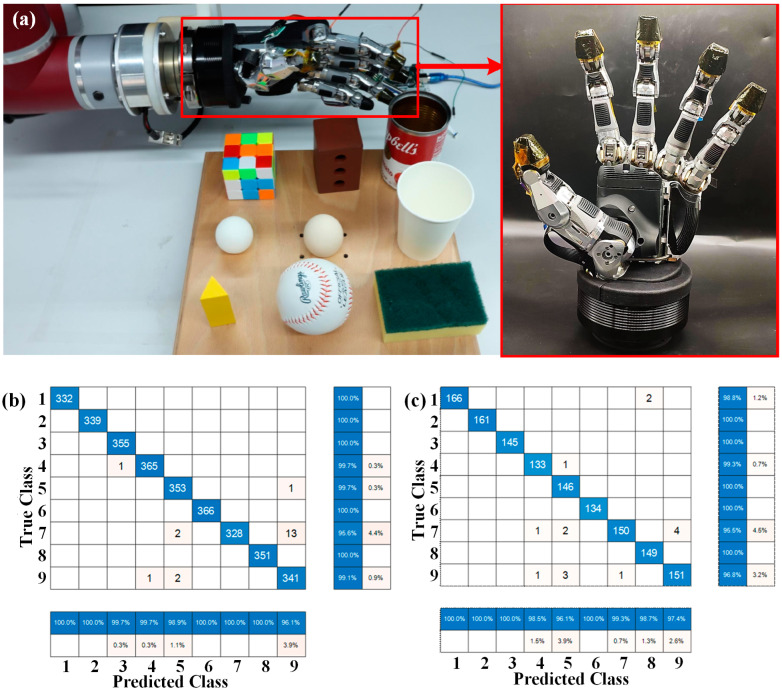
(**a**) The diagram of the tactile perception system of the SCHUNK five-fingered manipulator with the integrated tactile sensors; (**b**) the confusion matrix of the true and predicted classes of the training data; (**c**) the confusion matrix of the true and predicted classes of the test data.

Recognizing the unknown grasped object is significant in delicate operations that rely on the detection and reconstruction of the shape and modulus of objects. Currently, object recognition is typically performed by combining different technologies, such as vision, ultrasound, and laser technologies. However, in a low-visibility environment, for instance, at night or under the smoke, the tactile detection of a dexterous hand could be more important. In addition, the grasped object might be damaged if the wrong control strategy is employed in the grasping control system. Therefore, it is necessary that a robotic dexterous hand tactile system can obtain real-time contact information, which can assist in completing the classification and recognition of the grasped object. By integrating the proposed tactile sensor array on the SVH 5-finger servo-electric gripping hand (SCHUNK SE & Co. KG, Lauffen, Germany), grasping operations on a variety of objects can be conducted. To realize the recognition and classification of grasped objects, detection data on the five-finger sensor units during the grasping process were recorded, trained, and learned, as shown in Figure 10a. A total of nine different objects were selected, as listed in Table 2; among them, a sponge, a ping-pong ball, a paper cup, and an egg represented lightweight and fragile objects. During the experiment, the robotic dexterous hand completed the grasping operation in the first 10 s (pre-grasp for 5 s and grasp for 5 s) and was released after 20 s (keeping grasp for 10 s). For the tactile perception system on the dexterous hand, the tactile data were recorded and transmitted to the upper device with a frequency of 50 Hz by the customized measurement circuit. Due to the slip in the beginning of the object grasp operation, more datapoints were recorded and considered in the data training, including the dynamic response in the initial contact and the static performance during the stable grasp of the tactile perception system. To ensure the accuracy of the grasping information, each object was grasped six times. A three-layer backpropagation (BP) neural network structure was used to identify and classify the nine objects. Since the robotic dexterous hand had a total of 125 tactile sensor units, the sampling data of the tactile sensor units and the number of input-layer neurons were both set as 125. Since the output data of the BP neural network could be only zero or one, when it was used for classification, the number of the output layer nodes was set to nine. The number of hidden layer nodes can be determined by the following empirical formula:

(5) $$h=\sqrt{m+n}+a\;$$

where *h* is the number of hidden layer nodes; *m* and *n* are the number of input and output nodes, respectively; and *a* is the adjustment constant, which usually has a value of 1–10. The contact force values of each tactile unit at different stages of the grasping process are recorded in the grasp experiment, and 70% and 30% of the overall data are used as training samples and test samples, respectively. The mean square errors of the training data samples and test data samples with different numbers of hidden layer nodes were calculated. When the number of hidden layer neurons is set as 13, a relatively higher calculation accuracy has been achieved.

In order to reduce the test error, the “mapminmax” function is selected to normalize the input data, and the normalization interval is [0, 1]. The hidden layer selects the “tansig” tangent function, and the output layer selects the “purelin” linear function. The maximum number of iterations, the target training set error threshold and the learning rate were set as 1000, 10^−6^, and 0.01, respectively. In addition, the BP neural network test error was calculated. According to the calculation, the classification prediction accuracy of the BP neural network for the grasped objects in the training set is 99.36%, and the classification prediction accuracy for the grasped objects in the test set is 98.88%. The results show that the BP neural network can achieve the classification and recognition function of the grasped objects with a high classification accuracy.

The confusion matrices of the true and predicted classes of the training and test data are presented in Figure 10b and Figure 10c, respectively. The results indicated that in the training data, when the true class was 7 (i.e., the ping-pong ball), the prediction accuracies for the training and test data were 95.6% and 95.5%, respectively, and they were both lower than that of the objects in other categories. Thirteen training data samples and four test data samples were classified as category 9 (i.e., egg). This was due to the similar shapes of the egg and the ping-pong ball, and there were fewer points of contact with the tactile sensor units during the grasping process of these two round objects. In addition, in the egg-releasing process, due to the reduction in the grasping force, the real-time data from each tactile sensor unit were close to the real-time data when grasping the ping-pong ball at the stable stage, resulting in an incorrect classification result. According to the confusion matrix of the classification results of the training and test data, the classification accuracy of the nine objects was higher than 95%.

In summary, the recognition and classification of grasped objects by the dexterous hand could be conducted by the proposed tactile sensing array with high repeatability. Our sensor can enable the implementation of challenging tasks in robotics, such as reliable grasping and object recognition in a low-visibility environment.

## 6. Conclusions

Considering the tactile detection requirements for robotic dexterous hands, in this study, a flexible tactile sensor unit with high repeatability is proposed. In the proposed sensor design, a supporting layer is introduced to eliminate the initial value drift of the tactile sensor. The proposed sensor is examined through finite element simulations and experimental tests, and the characteristics of the proposed tactile sensor are investigated. The detection range of the proposed tactile sensor is 0–5 N, and its resolution is 0.2 N; the repeatability error of the proposed tactile sensor can be reduced to 1.5%. In addition, the response times under the loading and unloading conditions are 80 ms and 160 ms, respectively. Moreover, a 3D force detection method by decoupling non-coplanar tactile sensor units is developed. The preliminary object hardness recognition and the classification of objects with different shapes and categories are conducted. Through the use of the BP neural network, the classification and recognition of nine types of objects are realized, and a classification accuracy higher than 95% is achieved. The results show that the proposed tactile sensor array with high repeatability could be beneficial to delicate manipulation for robotic dexterous hands.

## Figures and Tables

**Table 1:** The parameters of the finite element modeling of the flexible tactile sensor unit.

Component	Dimensions	Elastic Modulus	Poisson Ratio
Cover layer	5 mm × 5 mm × 0.02 mm	2.9 GPa	0.370
Supporting layer	Rectangular: 5 mm × 5 mm × 0.15 mm Hollow: Φ2.5 mm × 0.15 mm	2.0 MPa	0.480
Sensing layer	Φ2.5 mm × 0.1 mm	2.2 MPa	0.473
Electrode layer	5 mm × 5 mm × 0.09 mm	200 GPa	0.300

**Table 2:** The object types and their assigned classes in the object classification process performed by the BP neural network.

Object Type	Class
Baseball	1
Cylinder block	2
Rectangular block	3
Rubik’s cube	4
Triangular prism	5
Sponge	6
Table tennis	7
Paper cup	8
Egg	9

## Data Availability

The original contributions presented in this study are included in the article. Further inquiries can be directed to the corresponding author.
